# Supplementary material for: Identification of photoperiod-induced specific miRNAs in the adrenal glands of Sunite sheep (Ovis aries)
Source: Front Vet Sci. 2022 Jul 22;9:888207. doi: 10.3389/fvets.2022.888207 (PMC9354845; doi:10.3389/fvets.2022.888207)
Supplement: Supplementary file 1 [file Data_Sheet_1.ZIP › Supplementary file/miRDeep2 file.zip!/miRDeep2 file/Know_Structure/SP42A2/oar-mir-381.pdf]

miRBase precursor : oar-mir-381
Total read count : 1354
oar-miR-381-5p read count : 0
oar-miR-381-3p read count : 1354
remaining reads : 0

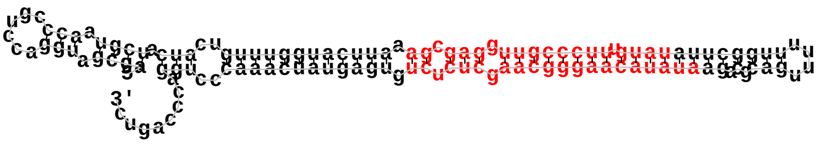

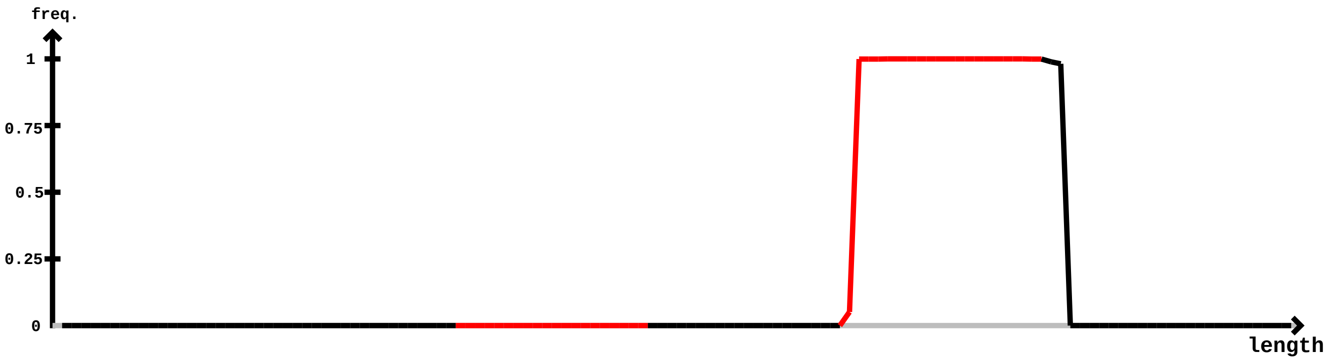


```
                                                                                 oar-miR-381-3p
                                         oar-miR-381-5p
5'- agcgauggaccugcccaaugcuacuacuguuugguacuuaaagcgagguugcccuuuguauauucgguuuuuugacgcagaauauacaagggcaagcucucugugaguaucaaacccuggacccaguc  -3'   exp
    ((((.(((......))).)))).(((..((((((((((((.((.(((.((((((((.((((((((((((....))).).)))))))))))))))).))).)).))))))))))))..)))........   reads   mm
    .................................................................................auauacaagggcaagcucucu..........................   2       0
    .................................................................................auauacaagggcaagcucucug.........................   1       0
    .................................................................................auauacaagggcaagcucucugu........................   66      0
    ..................................................................................uauacaagggcaagcucu............................   1       0
    ..................................................................................uauacaagggcaagcucucu..........................   12      0
    ..................................................................................uauacaagggcaagcucucug.........................   9       0
    ..................................................................................uauacaagggcaagcucucugu........................   1262    0
    ......................................................................................acaagggcaagcucucugu........................   1       0
```
